# Health care help seeking behaviour among prisoners in Norway

**DOI:** 10.1186/1472-6963-11-301

**Published:** 2011-11-04

**Authors:** Merete Berg Nesset, Åse-Bente Rustad, Ellen Kjelsberg, Roger Almvik, Johan Håkon Bjørngaard

**Affiliations:** 1 St.Olav's University Hospital, Division of Psychiatry, Forensic Dept. Brøset, Centre for Research and Education in Forensic Psychiatry, 7440 Trondheim, Norway p.o.box 1803 Lade, 7440 Trondheim, Norway; 2 Centre for Forensic Psychiatry, Oslo University Hospital, 0407 Oslo, Norway; 3 University Hospital of Northern Norway, dept. of Substance Use and Specialized Psychiatric Services, Tromsø, Norway; 4 Norwegian University of Science and Technology, Dept. of Public Health and General Practice, 7465 Trondheim, Norway

## Abstract

**Background:**

Prisoners are associated with high health care needs compared with the general population. This study aims to investigate prisoners' use of health service.

**Methods:**

A cross-sectional study of 29 prisons in central and southern parts of Norway. A questionnaire was distributed to 1, 454 prisoners (90% response rate). Multilevel analyses were employed to analyse help seeking behaviour among the prisoners.

**Results:**

Help seeking was substantially associated with sleep problems and drug problems. There was also a tendency for closed prisons as well as high staffing levels of healthcare professionals to be associated with elevated health care use.

**Conclusions:**

This study suggests that sleep problems and drug use are most frequently associated with health service use. The differences in health care use between prisons suggest that the implementation of prison health care standards should be addressed.

## Background

There is strong evidence that incarcerated people have higher rates of mental and physical illness and have a higher risk of suicide than the general population [1,2]. Prisoners have higher levels of anxiety, sleep problems and depression than the general population [2,3]. Furthermore, comorbidity of mental health problems and substance misuse disorders is common [4,5]. Furthermore, communicable diseases such as HIV and hepatitis B infections are especially prevalent [3]. Asthma and chronic obstructive pulmonary disease are also among the most frequent physical disorders [6]. Despite high morbidity, it is suggested that prisoners underuse health services [7,8] and that they are characterised by infrequent use of health care services when they are not in prison [3]. Distrust of health care professionals and lack of awareness of available services have been suggested in earlier studies as explanations for low help seeking [7,9]. In 2007, Hanratty and colleagues recommended that in order to assess the availability of a health service across socioeconomic groups, one should consider it in relation to the different groups' level of health needs [10].

As in other countries with universal health care systems, the health care system for Norwegian prisoners is integrated into the general health services for the community, but may be situated inside the prison. Hence the prisoners are not able to choose which health care provider they prefer. Bjørngaard and colleagues [11] found in a survey in 2009 that prisoners were dissatisfied with the prison health services, in contrast to findings from most satisfaction studies [12]. There seems to be a lack of consensus in the criminal justice system about how and when to assess prisoners' mental health [13]. Not knowing who to address their problems to has been highlighted as a barrier to help seeking [14]. Also, prisoners have reported concerns about the professional service provider as a barrier to seeking help while imprisoned [15].

### The aim of the present study

There is sparse knowledge of why prisoners seek help for health problems [14]. We therefore need to know more about the ability of the health service to meet the needs of different groups of prisoners in an effective manner. In this study, we wanted to address current limitations in this area by investigating the following research questions:

1. What is the association between the mental and physical health, drug misuse and sleep problems of prisoners and the prevalence of health care service use?

2. To what extent do socioeconomic status and demographic variables such as gender, age and ethnicity affect use of health care services?

3. To what extent are there differences between the prisons in the level of contact with the prison health service?

4. Are structural characteristics of the prison health care services associated with the prevalence of health care service use?

## Methods

### Norwegian prison health services

Norwegian prison health services have been an integrated part of the national public health service since 1988, when there was a transfer of responsibility from the Ministry of Justice to the Ministry of Health and Care Services. The main objective was to ensure that prisoners would receive the same health service as the general population. The prison health service is run by the municipal and the county health administration, with all expenses for primary health care being covered by the state. Norway also has four state-owned health authorities. The health authority where the prison is situated is responsible for the specialist services, for which it receives earmarked financial support from the state. Large prisons have a full-time health care service inside the prison whereas smaller prisons have a part-time service. If necessary during imprisonment, prisoners will be escorted to general psychiatric or somatic hospitals for treatment. Violent offenders who are psychotic at the time of the index offence should be sentenced to psychiatric care, often in high or medium secure psychiatric hospitals. They would consequently be diverted from the correctional system.

### Data collection procedures and participation rates

The study was approved by the Regional Committee for Medical Research Ethics, Region East. Information about inmates' help seeking behaviour was obtained from all prisons in the Central and Southern regions of Norway (N = 29) in 2007. The prisons included in the study represented 62% of the total prison capacity in Norway and was considered to offer a representative cross-section of the prison population. Except in one prison, data were collected by two experienced psychiatric nurses who contacted the inmates personally and helped them complete a questionnaire, either individually or in group meetings. In the last prison, the prison staff distributed and collected the forms. The questionnaire (the English version is available in additional file 1) was developed in Norwegian and translated into English and German; of the 1454 respondents, 90% completed the Norwegian version, 9% the English version and 1% the German version.

All 29 prisons in the central and Southern regions of Norway were asked to participate in the study. At the time of the survey, these held a total of 1955 prisoners. Of these, 131 were not present, 31 had already participated in the survey at another prison, 93 were excluded due to language problems, 5 were excluded due to reading/writing disabilities, 48 were in solitary confinement or were ill, and 28 were unavailable because of previously arranged visits (involving lawyers, doctors, or family members). In total, 1619 prisoners were thus considered eligible. Of these, 123 declined to participate and another 42 did not participate for reasons unknown, leaving a sample of 1454 prisoners (90% of those eligible for the survey).

### Independent variables

Self-reported health was hypothesized to be a major factor in prisoners' help seeking. Self-reported health was assessed with two 5-integer scales (mental and physical health) ranging from excellent to poor health. Drug use (5-integer scale ranging from no use to all the time) and sleep problems (5-integer scale ranging from no problems to all the time) was included as it could influence prisoner's wish for prescribed medication and also be associated with health status. Variables such as age (continuous scale), sex, education (compulsory, secondary or college/university), ethnicity/language (Norwegian, European or Non-European as first language), and type of sentence (serving sentence, remand prisoner or preventive detention) were included because we assumed that they might be related to help seeking behavior, and to adjust for compositional differences across prisons. Furthermore, we hypothesized that the number of full time health care workers per 100 inmates (continuous scale), the prison size (continuous scale) and the prison type (open, closed or mixed) could influence the availability of the services. All models were adjusted for the length of imprisonment (continuous scale) for adjustment for differences in composition across prisons.

Descriptive statistics are summarised in Table 1.

**Table 1:** Descriptive statistics of study participants and prisons

Variables	N	%	Mean (SD)
***Patient level variables***			

Contact with prison health services			
Yes	945	66	
No	480	34	
Age	1, 433		34.7(10.7)
Sex			
Female	78	5	
Male	1, 376	95	
Drug misuse outside prison			
No	706	50	
Rarely	126	9	
Sometimes	251	18	
Often	160	11	
All the time	174	12	
Sleep problems			
No	280	20	
Rarely	166	12	
Sometimes	393	27	
Often	298	21	
All the time	293	20	
Physical health			
Excellent/very good	460	32	
Good	570	39	
Not so good	251	17	
Poor	166	12	
Mental health			
Excellent/very good	493	34	
Good	415	29	
Not so good	289	20	
Poor	242	17	
Education			
Compulsory/none	524	37	
Secondary education	642	45	
University/college education	255	18	
First language			
Scandinavian	972	68	
European	221	15	
Non-European	237	17	
Type of imprisonment			
Serving sentence	1, 113	77	
Remand prisoner	284	19	
Preventive detention	54	4	
***Prison level variables***			
Full time staff equivalents/100 prisoners	29		3.2(1.6)
Capacity (number of prisoners)	29		71.2(77.5)
Prison type			
Open prison	9	31	
Closed prison	15	52	
Mixed prison	5	17	

### Statistical procedures

The material was divided into two hierarchical levels - prisons and prisoners - and multi-level regression analysis was performed with the statistical program Stata [16]. The dependent variable (present contact with prison health services or not) was dichotomous, and logistic regression analysis was performed.

The regression intercepts were allowed to vary randomly across prison health services. We estimated a *conditional intraclass correlation coefficient *(ICC) - suggested for multi-level logistic regression models [16] where *U_j _*in the equation is the between-ward variance:

$$ICC=\frac{U_{j}}{U_{j}+\pi^{2}∕3}$$

For the present study the ICC gives an estimate of the relative importance of the prison level for the individuals' propensity for use of prison health services.

We first analysed the variance attributable to differences between prison health services, only adjusting for length of imprisonment (Table 2). Next, we analysed each independent variable's association with the dependent variable ('unadjusted' columns in table 2). Lastly, we entered all independent variables in the fully adjusted model ('adjusted' columns in table 2). A 95% confidence interval was used for the parameter estimates.

**Table 2:** Multilevel logistic regression analysis of contact with the prison health services in 29 prisons

Independent variables	**Unadjusted OR**^**1**^	95% CI		**Adjusted OR**^**1**^	95% CI	
***Patient level variables***						
Age in 10-year intervals	1.24	1.1	1.4	1.26	1.1	1.4
Sex (female = 1)	0.84	0.4	1.8	0.76	0.3	1.9
No drug misuse outside prison (reference)	1.00			1.00		
- rarely	1.15	0.8	1.8	1.15	0.7	1.8
- sometimes	1.21	0.9	1.7	1.26	0.9	1.8
- often	1.06	0.7	1.6	0.98	0.6	1.5
- all the time	1.87	1.3	2.8	1.65	1.1	2.6
No sleep problems (reference)	1.00			1.00		
- rarely	1.44	0.9	2.2	1.29	0.8	2.0
- sometimes	1.46	1.0	2.1	1.26	0.9	1.8
- often	2.93	2.0	4.3	2.31	1.5	3.5
- all the time	2.83	1.9	4.2	1.96	1.3	3.1
Excellent/very good physical health (reference)	1.00			1.00		
- good	1.05	0.8	1.4	0.88	0.6	1.2
- not so good	1.66	1.2	2.4	1.00	0.7	1.6
- poor	1.93	1.2	3.0	1.09	0.6	1.9
Excellent/very good mental health (reference)	1.00			1.00		
- good	1.09	0.8	1.5	0.93	0.7	1.3
- not so good	1.87	1.3	2.6	1.31	0.9	2.0
- poor	2.10	1.5	3.1	1.45	0.9	2.3
Compulsory/no education (reference)	1.00			1.00		
- secondary education	0.89	0.7	1.2	0.97	0.7	1.3
- university/college education	0.75	0.5	1.1	0.75	0.5	1.1
Norwegian as first language (reference)	1.00			1.00		
- European	0.95	0.7	1.3	1.16	0.8	1.7
- non-European	1.08	0.8	1.5	1.13	0.8	1.6
Serving sentence (reference)	1.00			1.00		
- remand prisoner	0.68	0.5	1.0	0.60	0.4	0.9
- preventive detention	0.72	0.3	1.8	0.45	0.2	1.2
						
***Prison level variables***						
Full time staff equivalents/100 prisoners	1.14	1.0	1.3	1.09	0.9	1.3
Capacity (number of prisoners)	1.00	1.00	1.00	1.00	1.0	1.0
Open prison (reference)	1.00			1..00		
- closed prison	2.13	1.3	3.5	1.95	1.0	3.9
- mixed prison	1.32	0.7	2.4	1.25	0.6	2.5
						
Prison-level variance	0.28	0.1	0.6	0.20	0.1	0.5
ICC	0.08			0.06		
N	1, 383(max)			1, 291		

^1^Also adjusted for length of imprisonment

## Results

### Descriptive statistics

About 66% of the responding inmates were currently users of prison health services, 95% were males, and the mean age was 35 years. Mental health problems and sleep difficulties was clearly associated (Spearman's rho = 0, 44), as well as sleep difficulties and physical problems (Spearman's rho = 0, 28). Mean length of imprisonment was 400 days (SD:727), with a median of 125 days. There were substantial differences between prisons in length of imprisonment (ICC = 0, 25 - estimated with an empty linear multilevel model).

### Results of the multilevel regression analyses

Table 2 shows the results of the multilevel regression analysis. The ICC in the unadjusted model was 0.08 (p < .01), and this was reduced to about 0.06 in the full model (p < .01).

Age was positively associated with contact - 26% higher odds per 10-year increase in age (CI 1.1-1.4), while the difference between male and female prisoners was small. Respondents who reported the highest level of drug misuse when not in prison were more likely to be in contact with the prison health services than those not taking drugs.

Physical, mental and sleep problems were clearly associated with contact in the unadjusted models. In the adjusted model, the association between service use and physical and mental health was attenuated.

The level of contact did not substantially differ between education groups - however there was a tendency towards less contact for the highest education group, but the result was uncertain with a wide confidence interval (CI 0.5-1.1). The level of contact did not differ according to having a native language other than Norwegian. The fully adjusted model indicated a tendency towards less contact for prisoners in preventive detention and on remand compared with those serving sentences.

The level of contact was not strongly associated with the size of the prison. There was a tendency towards more contact with increasing staffing levels. Prisoners in closed prisons were also more likely to be in contact with health services.

## Discussion

This study of help seeking behaviour by Norwegian prisoners indicated a prevalence rate of health service use at about 66% of the prison population. Sleep problems were highly associated with use of prison health services. Higher age and drug use when not imprisoned also promoted help seeking. The type of imprisonment influenced health service use: those in preventive detention or on remand had less contact with health care professionals than those serving sentences. There was a tendency for higher levels of prison health care staffing and closed prisons to be associated with increased contact. When only mental and physical health problems were taken into account, there was a tendency towards increased help seeking behaviour. In the adjusted model, however, the influence of mental health and physical disorders was clearly attenuated, which may indicate that the prisoners with mental and physical health problems did not seek professional help unless they had a co-occurring problem - for instance, sleep difficulties or drug use. This assumption is supported by the clear associations between mental and physical problems and sleep problems.

Kjelsberg and Hartvig [6] found that 35% of the prison population had psychiatric disorders. Hence, sleep problems might be a symptom that is specific and easier to seek help for, and a marker for more serious underlying problems related to mental health and/or drug use. New Zealand prisoners were often unwilling to seek health care help when suicidal [8]. Moreover, Pratt and co-workers [1] found that during the first year after discharge from prison, prisoners were at elevated risk of suicide compared with the general population. The suicide risk was especially high during the first 28 days after release from prison. Howerton and colleagues [7] reported several factors influencing help seeking behaviour among male offenders. Fear of a mental illness diagnosis was presented as one explanation for not seeking help for mental health problems. Given the high prevalence of prisoners' mental health problems shown in previous research, health care workers should pay attention to prisoners who present sleep- and/or drug-related problems as the reason for contact.

Age was positively associated with help seeking in our study, supporting previous research suggesting a relationship between help seeking behaviour and age [8,9]. Possible explanations are that older prisoners are more aware of their own dependencies and problems than younger prisoners are and, older prisoners' help seeking behaviour may reflect increased health needs simply because of their higher age [2].

Prisoners in preventive detention or on remand had less contact with health care professionals than those serving sentences. On the individual level, one might speculate that these inmates would mistrust anyone associated with the prison system, either because of adverse childhood experiences leading to a general distrust of others or as a result of previous negative experiences with health care professionals[7]. However, less use of health care services might also be an indication of poor availability of the service to these inmate subgroups.

In the light of the often low socioeconomic status and high health care needs of prisoners, the requirement for good access to prison health care service is clear [3,13]. There were substantial differences between prisons in our study in the prevalence of health service use. We did not find any association between inmates' help seeking behaviour and the size of prison, but we found some differences between open and closed prisons. There was also a tendency for inmates to seek help more often in prisons with higher health care staffing. The findings in our study support existing knowledge that there are organisational differences between prisons and that efforts invested in prison health care are significant for prisoners' health care use [9,11,13]. An underlying conflict of interest between views of prisons as places for punishment or for rehabilitation has been suggested as a reason for the differences between prisons, indicating arguments of idealistic and cultural dimensions [13]. Our findings support earlier research on this issue indicating the need for attention to how the health care system in prisons is organised and made available to the prisoners [15].

In this study, neither education nor ethnicity appeared to contribute to explanations of differences in prisoners' help seeking behaviour. Consequently, our findings did not support earlier research indicating associations between education, ethnicity and help seeking behaviour [5,8,10]. However, similar findings were reported in a recent study of 177 prisoners' help seeking behaviour in connection with mental health problems, where no substantial relationship between ethnicity or qualification level and intentions to seek help was found [9]. In the light of indications that the incidence of mental health disorder among ethnic minority prisoners is higher than in the general prison population [5], more in-depth research on help seeking behaviour among ethnic minority inmates across countries would seem appropriate.

There were no substantial differences between the help seeking behaviour of male and female inmates in this study, but the result was uncertain probably as a consequence of few female inmates. Earlier research has indicated a higher prevalence of mental and somatic problems among female inmates [2,6], so that higher levels of help seeking behaviour in the female prison population would be expected. However, existing health promoting programmes are mainly designed for male prisoners and thus might not be pertinent to women [2]. Consequently, women may not be confident that the health care professionals are able to help them when they are experiencing difficulties and may thus turn to cell mates or family members for help [7-9].

The present study was dependent on a self-report measure for the assessment of health service use. Self-reports may be susceptible to several self-presentational biases, including denial or exaggerations of problems and the wish to present oneself in a positive manner. These biases are likely to be particularly relevant in prison settings, where the inmate may fear the consequences of truthful responses. However, we used a simple measure of contact with health service use and fear of consequences was thus unlikely to be a problem. Despite a high response rate in this study (90%), 93 prisoners were excluded because of insufficient ability to read or write Norwegian, English or German. Hence caution should be exercised in interpreting the finding that ethnicity was not a contributing factor in the explanation of help seeking behaviour.

To be able to provide good health care services, it is essential to start with the identification of prisoners in need of health care help [13]. In addition to structuring the health care process for the individual prisoner, there is a need for further debate on the issue of "care" versus "custody" that has been highlighted in the literature [13]. This study is cross-sectional, and the potential for identifying causal explanations of the findings regarding differences between prisons is limited. The study may however inspire more in-depth research on prison health services at the organisational level as well as at the prisoner level, with a more specific focus on addressing and meeting prisoners' diverse needs.

## Conclusions

Prisoners are associated with high health care needs compared with the general population. This study suggests that sleep problems and drug misuse are most frequently associated with health service use. Also, the clear association between sleep problems and mental health problems found in this study indicate that sleep disorders need more attention in prison health care. Further, higher staffing levels promote prisoners use of health care services. There are also indications that prisoners in closed prisons perceive the health care service as more available. There is currently a lack of studies that address the implementation of health care standards and guidelines in practice [13]. Such studies might provide answers regarding the differences shown in prisoners' perceived satisfaction with the health service [11], as well as their actual health service use across prisons.

## Competing interests

The authors declare that they have no competing interests.

## Authors' contributions

MBN drafted the manuscript. ÅBR, EK and JHB designed the study and developed the instrument. ÅBR carried out the survey in the prisons. JHB performed the data analysis and supervised the progress of the paper. All authors participated in reading and writing the manuscript as well as approved the final manuscript.

## Pre-publication history

The pre-publication history for this paper can be accessed here:

http://www.biomedcentral.com/1472-6963/11/301/prepub

## Supplementary Material

Additional file 1 **"What is your opinion about the prison health services?"**. A user satisfaction questionnaire concerning prison inmates' opinion about the prison health services. Click here for file
