# Supplementary material for: Health care help seeking behaviour among prisoners in Norway
Source: BMC Health Serv Res. 2011 Nov 4;11:301. doi: 10.1186/1472-6963-11-301 (PMC3221636; doi:10.1186/1472-6963-11-301)
Supplement: Additional file 1 — "What is your opinion about the prison health services?". A user satisfaction questionnaire concerning prison inmates' opinion about the prison health services. [file 1472-6963-11-301-S1.DOC]

**What is your opinion about**

**the prison health services?**

**A user satisfaction study conducted by**

**The Centre for Research and Education in Forensic Psychiatry**

**in Oslo**

- *Tick off the answer that best expresses your opinion.*
- *If there are some questions you don’t feel like answering, then leave them open. But please answer the rest!*
- *This study does not include the dental services.*

**Part 1: General questions**

**Sex** □ Male □ Female

**Age**  ______ years

**Prisoner category** □ Remanded □ Serving sentence □ Preventive detention

**How many times have you been to prison before this time?** ___________

**If you are serving time now, how long is your present sentence?** _________________

**How long have you been in prison hitherto** (this time)? ___ years, ___ months, ___ days

**All told, would you consider your physical health as**:

| Perfect  □ | Very good  □ | Good  □ | Not so good  □ | Poor  □ |
| --- | --- | --- | --- | --- |

**All told, would you consider your mental health to be:**

| Perfect  □ | Very good  □ | Good  □ | Not so good  □ | Poor  □ |
| --- | --- | --- | --- | --- |

**What kind of health problems do you have?**

| Physical  only  □ | Mental  only  □ | Both physical and mental  □ |  | |  |  | | I do not have any health problems  □ |
| --- | --- | --- | --- | --- | --- |

**Do you find your physical health better or worse now, compared with before you were imprisoned**?

| Much  better  □ | A little  better  □ | Neither better  nor worse  □ | A little  worse  □ | Much  worse  □ |
| --- | --- | --- | --- | --- |

**Do you find your mental health better or worse now, compared with before you were** imprisoned?

| Much  better  □ | A little  better  □ | Neither better  nor worse  □ | A little  worse  □ | Much  worse  □ |
| --- | --- | --- | --- | --- |

**Do you use illegal drugs while not in prison**?

| Rarely  □ | Yes,  sometimes  □ | Yes,  often  □ | Yes,  all the time  □ | |  |  | | No, I do not use  illegal drugs  □ |
| --- | --- | --- | --- | --- | --- |

**Do you have sleep problems**?

| Rarely  □ | Yes,  sometimes  □ | Yes,  often  □ | Yes,  all the time  □ | |  |  | | No, I do not have  sleep problems  □ |
| --- | --- | --- | --- | --- | --- |

**Which is the highest educational level you have completed**?

| No school completed  □ | Elementary  school  □ | High-school  □ | College or university, less than 4 years  □ | College or university,  4 years or more  □ |
| --- | --- | --- | --- | --- |

**Which language did you first learn to speak**?

| English  □ | Another European language  □ | An Asian  Language  □ | An African  language  □ | Any  other  □ |
| --- | --- | --- | --- | --- |

**What is your country of citizenship**?

| Norway  □ | Another European  country  □ | An Asian country  □ | An African country  □ | A South American country  □ | A North American country  □ | Australia  □ |
| --- | --- | --- | --- | --- | --- | --- |

**Part 2: Your contact with the prison health services**

**Are you presently in contact with the prison health services?**

□ Yes □ No

**Did you use to be in contact with the health services, but are not presently so?**

□ Yes □ No

**Would you like to get in touch with the health services, but have not succeeded in this?**

□ Yes □ No

**If you have not succeeded in getting in touch with the health services, why do you think this is so?** *(You may tick off more than one answer, if you find that this applies to you)*

□ I have not needed to get in touch with the health services

□ I have just arrived. It seems reasonable that they haven’t had time to see me yet.

□ I have not been very explicit in stating my wish to see the health services

□ I believe the people from the health services have been slow in getting in touch with me.

□ I do not believe that the health services are able to help me.

□ I do not trust the health service people.

□ Other reasons. Explain why you believe you have not had any contact with the health services:

__________________________________________________________________________

__________________________________________________________________________

__________________________________________________________________________

**Are there people in the prison, other than those belonging to the health services, that you feel have been able to help you with your health needs?** *(Tick off more than one answer, if applicable)*

| Prison officer  □ | Prison priest  □ | Teacher  □ | Librarian  □ | Social worker  □ | Other  inmates  □ | Other than these  □ |
| --- | --- | --- | --- | --- | --- | --- |

**If you NEVER have been in contact with the prison health services, you have now completed the questionnaire.**

***THANK YOU for your time and effort!***

***IMPORTANT!!***

**You who PREVIOUSLY were or PRESENTLY are in contact with the prison health services, must complete the rest of the questionnaire too!!**

**Part 3: About your contact with the health services**

**Which part of the health services have you been in contact with**?

| The primary health service □ | The psychiatric health service  □ | Do not know which one  □ |
| --- | --- | --- |

**Which professions have you been in contact with**? (Tick off those applicable)

| Regular  nurse  □ | Psychiatric  nurse  □ | Regular doctor  □ | Psychiatrist  □ | Psychologist  □ | Physio-therapist  □ | Other profession  □ | Do not know  □ |
| --- | --- | --- | --- | --- | --- | --- | --- |

**All in all, how satisfied are you with the prison health services?**

| Very dissatisfied  □ | Quite dissatisfied  □ | So-and-so  □ | Quite satisfied  □ | Very satisfied  □ |
| --- | --- | --- | --- | --- |

**Have you received the help you think you need, regarding your physical complaints**?

| No,  absolutely not  □ | No, not good enough  □ | So-and-so  □ | Yes,  fairly good  □ | Yes,  absolutely  □ |
| --- | --- | --- | --- | --- |

**Have you received the help you think you need, regarding your mental health complaints**?

| No,  absolutely not  □ | No, not good enough  □ | So-and-so  □ | Yes,  fairly good  □ | Yes,  absolutely  □ |
| --- | --- | --- | --- | --- |

**Did you have to wait to get an appointment with the health services?**

| No  □ | Yes, but not long  □ | Yes, quite long  □ | Yes, far too long  □ |
| --- | --- | --- | --- |

**For how long have you had contact with the health services**?

| Less than 1 month  □ | Between 1 and  6 months  □ | Between 7 and 12 months  □ | Between 12 months and 3 years  □ | More than  3 years  □ |
| --- | --- | --- | --- | --- |

**How often during the last 3 months have you been in contact with the health services?** (Please answer even if you have been in contact with them less than 3 months)

| Only once  □ | 2 to 5 times  □ | 6 to 12 times  □ | More than 12 times  □ |
| --- | --- | --- | --- |

**What is your opinion about the number of times you have been in touch with the health services**?

| Right number of contacts  □ | A bit too infrequent  □ | Way too infrequent  □ |
| --- | --- | --- |

**Have you been approached by the health service workers in a courteous and respectful manner**?

| No,  not at all  □ | No,  not really  □ | To a certain degree  □ | Yes,  generally  □ | Yes, most definitely  □ |
| --- | --- | --- | --- | --- |

**How have you benefited from talking with your therapist?**

| No  benefit  □ | Little  benefit  □ | Some  benefit  □ | Large  benefit  □ | Very large benefit  □ | |  |  | | Not applicable  □ |
| --- | --- | --- | --- | --- | --- | --- |

**To which degree have you benefited from your contact with the prison health service**?

| No  benefit  □ | Little  benefit  □ | Some  benefit  □ | Large  benefit  □ | Very large benefit  □ |
| --- | --- | --- | --- | --- |

**Do you get enough time to talk with the health worker**?

| No,  not at all  □ | Very  limited  □ | To a certain degree  □ | Yes,  generally  □ | Yes, most definitely  □ |
| --- | --- | --- | --- | --- |

**In your experience, does the health worker understand your situation**?

| No,  not at all  □ | Very  limited  □ | To a certain degree  □ | Yes,  generally  □ | Yes, most definitely  □ |
| --- | --- | --- | --- | --- |

**Do you find that the therapy is suited to your situation**?

| No,  not at all  □ | Very  limited  □ | To a certain degree  □ | Yes,  generally  □ | Yes, most definitely  □ |
| --- | --- | --- | --- | --- |

**Does the therapist follow up planned interventions**?

| No,  not at all  □ | Very  limited  □ | To a certain degree  □ | Yes,  generally  □ | Yes, most definitely  □ |
| --- | --- | --- | --- | --- |

**Have you been able to tell the health workers the things you consider important about your condition**?

| No,  not at all  □ | Very  limited  □ | To a certain degree  □ | Yes,  generally  □ | Yes, most definitely  □ |
| --- | --- | --- | --- | --- |

**To what extent have you been able to influence the choice of therapeutic intervention**?

| No,  not at all  □ | Very  limited  □ | To a certain degree  □ | Yes,  generally  □ | Yes, most definitely  □ |
| --- | --- | --- | --- | --- |

**What do you think about the information you have received regarding the different choices of therapeutic interventions**?

| Very poor  □ | Rather poor  □ | So-and-so  □ | Fairly good  □ | Very good  □ |
| --- | --- | --- | --- | --- |

**What do you think about the information you have received regarding your complaints and/or your diagnosis**?

| Very poor  □ | Rather poor  □ | So-and-so  □ | Fairly good  □ | Very good  □ |
| --- | --- | --- | --- | --- |

**Have you been able to exert influence upon your medication**?

| Not at all  □ | Very little  □ | Somewhat  □ | Yes, definitely  □ | Yes,  very much  □ | |  |  | | I do not use medication  □ |
| --- | --- | --- | --- | --- | --- | --- |

**How do you rate the information you’ve been given regarding side-effects of medication you have started using after you came to prison?**

| Very poor  □ | Quite poor  □ | So-and-so  □ | Quite good  □ | Very good  □ | |  |  | | I do not use medication  □ |
| --- | --- | --- | --- | --- | --- | --- |

**Has any prison health worker treated you in an impolite or disrespectful manner**?

| No, never  □ | Yes, once  □ | Yes, sometimes  □ | Yes, often  □ |
| --- | --- | --- | --- |

**Do you feel that you have been taken seriously by the health services**?

| Not at all  □ | Not very much  □ | Somewhat  □ | Fairly much  □ | Very much so  □ |
| --- | --- | --- | --- | --- |

**As a whole, how have you experienced the prison health services?**

| Much worse than expected  □ | Somewhat worse than expected  □ | As  expected  □ | Somewhat better than expected  □ | Much better than expected  □ |
| --- | --- | --- | --- | --- |

Are there other issues regarding the health services in the prison that you want to bring up? If so, write it down in your own words below.

***Thank you!***

___________________________________________________________________________

___________________________________________________________________________

___________________________________________________________________________

___________________________________________________________________________

___________________________________________________________________________

___________________________________________________________________________

___________________________________________________________________________

___________________________________________________________________________

___________________________________________________________________________

___________________________________________________________________________
